# Chemical Physical Characterization and Profile of Fruit Volatile Compounds from Different Accesses of *Myrciaria floribunda* (H. West Ex Wild.) O. Berg through Polyacrylate Fiber

**DOI:** 10.3390/molecules26175281

**Published:** 2021-08-31

**Authors:** Yesenia Mendoza García, Ana Luiza Coeli Cruz Ramos, Ana Cardoso Clemente Filha Ferreira de Paula, Maicon Heitor do Nascimento, Rodinei Augusti, Raquel Linhares Bello de Araújo, Eurico Eduardo Pinto de Lemos, Júlio Onésio Ferreira Melo

**Affiliations:** 1 Centro de Ciências Agrárias, Campus A. C. Simões, Universidade Federal de Alagoas, Rio Largo 57072-970, Brazil; jenny_thesiba@hotmail.com (Y.M.G.); eurico@ceca.ufal.br (E.E.P.d.L.); 2 Departamento de Alimentos, Faculdade de Farmácia, Campus Belo Horizonte, Universidade Federal de Minas Gerais, Belo Horizonte 31270-901, Brazil; analuizacoeli@gmail.com (A.L.C.C.R.); raquel@bromatologiaufmg.com.br (R.L.B.d.A.); 3 Departamento de Ciências Agrárias, Instituto Federal de Educação, Ciência e Tecnologia de Minas Gerais, Campus Bambuí, Bambuí 38900-000, Brazil; ana.paula@ifmg.edu.br (A.C.C.F.F.d.P.); maicon-naza@hotmail.com (M.H.d.N.); 4 Departamento de Química, Campus Belo Horizonte, Universidade Federal de Minas Gerais, Belo Horizonte 35702-031, Brazil; augusti.rodinei@gmail.com; 5 Departamento de Ciências Exatas e Biológicas, Campus Sete Lagoas, Universidade Federal de São João Del-Rei, Sete Lagoas 36307-352, Brazil

**Keywords:** Myrtaceae, native fruit, volatile compounds, sesquiterpenes

## Abstract

Among the many species of native fruit of Brazil that have been little explored, there is *Myrciaria floribunda* (also known as rumberry, cambuizeiro, or guavaberry), a species with significant variability, which has fruits of different colors (orange, red, and purple) when ripe. The physical-chemical characteristics evaluated were fruit weight (FW), seed weight (SW), pulp weight (PW), number of seeds (NS), longitudinal diameter (LD), transverse diameter (TD), format (LD/TD), hydrogen potential (pH), soluble solids (SS), titratable acidity (TA), and ratio (SS/TA); further, the volatile organic compounds (VOCs) of nine accesses of rumberry orchards were identified. The averages of the variables FW, SW, PW, NS, LD, TD, shape, and firmness were 0.76 g, 0.22 g, 0.54 g, 1.45, 10.06 mm, 9.90 mm, 1.02, 2.96 N, respectively. LD/TD data showed that the fruits have a slightly rounded shape (LD/TD = 1). The averages for pH, SS, TA, and SS/TA were 3.74, 17.58 Brix, 4.31% citric acid, and 4.31, respectively. The evaluated parameters indicated that the fruits can be consumed both in natura and industrialized, with the red-colored fruits presenting a good balance of SS/TA, standards demanded by the processing industries. Thirty-six VOCs were identified, with emphasis on the sesquiterpenes. Caryophyllene (21.6% to 49.3%) and γ-selinene (11.3% to 16.3%) were the most predominant compounds in rumberry fruits.

## 1. Introduction

*Myrciaria floribunda* (H. West ex Willd.) O. Berg is a species belonging to the Myrtaceae family, from which the fruits are popularly known as ‘camboim,’ ‘jabuticabinha,’ ‘myrtle,’ ‘duke,’ ‘goiabarana,’ ‘araçazeiro,’ ‘cambuí,’ and ‘rumberry.’ In Brazil, this species can be found mainly in the Atlantic Forest biome, easily observed in the Northeast of the country, specifically in the states of Sergipe and Alagoas [1]. 

The rumberry has excellent potential for commercial export, widely cultivated due to its edible fruits, consumed both in natura and industrialized. These are globose fruits, with a slightly acidic flavor, of varied color (orange, red, and purple) [2], with high sugar content and are rich in bioactive compounds (carotenoids, flavonoids, and phenolic acids) [3]. When ripe, the fruits become attractive because of the intense citrusy and slightly sweet aroma of the fleshy and succulent pulp [4]. 

Chemical compounds are responsible for the characteristic flavor of tropical or subtropical fruits, playing an important role in the quality of the fruits and their products, determined by several parameters, such as appearance, flavor, nutritional value, and food security [5,6]. However, the chemical composition of rumberry fruits is still unknown, which prevents further tapping into market potential. 

The acceptance of fruits is directly related to taste and aroma, released mainly during the ripening phase of the fruit. These parameters are among the most important quality criteria of fresh and processed foods, since the aroma and taste of the fruit provoke sensory sensations that stimulate the desire to consume it [7]. These sensations are attributed to hundreds or thousands of volatile and non-volatile substances that are present in food and which are measured by sensory organs. [8]. In general, the aromatic compounds of fruits are low-molecular-weight substances, partially soluble in water and volatile at room temperature, belonging to a wide variety of chemical families such as alcohols, esters, acids, aldehydes, ketones, aliphatic and aromatic esters, terpenes, hydrocarbons, and phenolic and sulfur compounds, which are found in different concentrations [9].

Recent studies have shown that the leaves of *M. floribunda* produce essential oils rich in monoterpenes (53.9%) and, among them, 1,8-cineole is the main constituent (38.4%) [10]. The essential oils of *M. floribunda* showed pharmacological potential in terms of insecticidal activity [10], inhibition of acetylcholinesterase [11], and antimicrobial and antitumor activity [12].

The determination of volatile compounds contributed to the discovery of a new aroma. For this, there are techniques such as solid-phase microextraction (SPME), which is based on the extraction and rapid concentration of volatile and semi-volatile organic compounds without the use of organic solvents [7]. In this type of extraction, the analyte is adsorbed on a silica fiber coated with a polymer, which is inserted in the headspace bottle for subsequent thermal desorption, injecting the analytes in a gas chromatograph (GC) that, coupled with mass spectrometry (MS), provides speed and practicality in the analysis of the volatile profile of the fruits. 

In the absence of studies evaluating the volatile profile of *Myrciaria floribunda* fruits using the solid-phase microextraction technique, the objective of the present work was to characterize the volatile profile of nine rumberry accesses through the polyacrylate fiber, using SPME-HS/GC–MS.

## 2. Results and Discussion

### 2.1. Physicochemical Characterization of Rumberry Fruits

Significant differences (*p* ≤ 0.05) were observed between the different accesses evaluated, for the physicochemical characteristics, except for pH (Table 1). The fruits had an average weight of 0.76 g, with the highest values found in AC132. These results agree with those reported by Silva et al. [13] in the evaluation of the biometry of fruits of the same species. In guabiju fruits (*Myrcianthes pungens*), the average weight was 2.87 g [14], and for *Campomanesia rufa*, 10.88 g [15], species belonging to the Myrtaceae family. 

The fruits of accesses AC67, AC92, and AC160 showed the lowest values in terms of fruit weight, pulp weight, and a number of seeds. The study by Rodrigues et al. [14] obtained an average for the weight of the fruit (2.87 g), pulp weight (1.29 g), and seed weight (0.49 g) higher than those reported in the present study, while the number of seeds remained equal, containing one to two seeds per fruit [1].

The accesses AC132 (11.64 mm of DL and 10.43 mm of DT) and AC156 (10.66 mm of DL and 11.50 mm of DT) presented the largest dimensions and the highest pulp yields (0.64 and 0.69 g, respectively). The fruits had a medium diameter (longitudinal and transversal) greater than those reported in red jambo (*Syzygium malaccensis*) [16] fruits and inferior to those reported for rumberry fruits (*Myrciaria floribunda*), in their orange and purple color—4.09 to 4.47 mm and 4.47 to 4.87, respectively [1].

The shape of the fruits showed an overall average of 1.02, characteristic of fruits with a more rounded shape (LD/TD = 1). The shape of the fruits is an index of ripeness since they are usually measured by the ratio of their diameter. This characteristic is also a factor of industrial quality since the industries prefer round-shaped fruits for easy cleaning and processing operations.

Statistically, there was not a significant difference regarding the firmness of the fruits. However, the fruits of the AC160 access showed greater firmness, with an average of 5.67 N, while the AC67 access showed the lowest average of 1.46 N. It is noteworthy that, to date, there are no reports in the literature regarding the firmness of rumberry fruits. 

According to Becker et al. [17], firmness is a very important quality attribute in fruits because the greater the firmness, the greater the resistance to mechanical injuries during transport and commercialization of fruits responsible for flavor and aroma. 

Rumberry fruits had a pH from 3.35 to 3.99, with an average value of 3.74, with no significant difference between the accesses studied (Table 1). These values were similar to those reported in fruits of the same species [1], whereas in fruits of the same genus, a pH of 2.41 was reported [18].

However, Almeida et al. [19] found pH values of 3.10 and 3.39 for the varieties of jabuticaba *Myrciaria jabuticaba* and *Myrciaria grandifolia*, respectively. According to the authors, the most acidic products are naturally more stable in terms of deterioration, and the relative proportion of organic acids present in fruits and vegetables might vary according to the degree of ripeness and growth conditions. This information is relevant for selecting accesses since more acidic fruits can be better used in the food industry.

The SS content varied significantly between accesses. AC67, AC92, AC132, and AC137 registered the highest values from 17.65 to 22.78 Brix, while accesses AC112, AC136, AC153, AC156, and AC160 registered values in a range of 13.25–16.88 Brix. These values were higher when compared to those reported by Vieira et al. [20] at different stages of fruit ripening of ubaia-azeda *(Eugenia azeda*), whose values ranged from 3.13 to 4.35 °Brix, as well as those reported by Souza, Silva and Aguiar [18] when obtaining values of 9.02 °Brix in ripe fruits of camu-camu *(Myrciaria dubia*).

As for AT, the lowest values found were in the AC137 (2.70), and AC160 (2.80) accesses. As for the accesses AC67, AC92, AC112, AC132, AC136, AC153, and AC156, there was no significant difference among them. Souza, Silva, Aguiar [18] reported values lower than those found when determining the chemical composition phases: unripe, semi-ripe, and ripe, finding averages of 2.75, 2.77, and 2.4 g of citric acid 100 g^−1^, respectively.

Seraglio et al. [21], when analyzing the physicochemical parameters of mature fruits of three species of Myrtaceae, reported values of 0.19 g of citric acid 100 g^−1^ for jabuticaba fruits *(Myrciaria cauliflora)*, 0.02 g for guabiju fruits (*Myrcianthes pungens*), and 0.04 g for jambolan fruits (*Syzygium cumini*), respectively.

TA and SS are a crucial reference point for the ideal level of fruit ripeness, in addition to being an important parameter for assessing the conservation status of all foods, once through the decomposition process (hydrolysis, oxidation or fermentation), which changes the concentration of organic acids, thus altering the acidity of the food [21].

The SS/TA ratio of rumberry fruits was higher than that reported by Vieira et al. [20] and Neto, Silva and Dantas [22], who found mean values of 2.45 and 2.98, respectively. Lattuada et al. [23] observed higher values, ranging from 13.12 to 17.10, in whole and ripe fruits of five samples of jabuticaba trees (*Plinia peruviana* and *Plinia cauliflora*).

For the fresh and/or processed fruit market, high SS values and SS/TA ratios are the most desirable and valued characteristics, both in natura and industrial use. In the case of consumers, they give preference to larger fresh fruits, with an appealing appearance, sweeter, and less acidic. Therefore, the best way to assess the fruit is through the SS/TA ratio, as it is more representative than the isolated analysis of SS or TA [6]. Thus, the fruits of the AC137 access would be the most suitable for presenting a higher SS/TA ratio (6.87).

### 2.2. Profile of Volatile Compounds

A total of 36 volatile organic compounds (VOCs) were identified by the polyacrylate fiber (PA) through solid-phase microextraction in the headspace mode (SPME-HS). The fruits of the different accesses of *Myrciaria floribunda* contained sesquiterpenes: 30.56% monoterpenes and 69.44% sesquiterpenes (Table 2).

In oils derived from leaves and flowers of *Myrciaria floribunda*, monoterpenes have been reported as the main components, with sesquiterpenes predominating in stem oil [11]. Regarding the lyophilized fruits of the same species, the characterization was different, as they presented a higher composition of monoterpenes (59.4%) than sesquiterpenes (40.6%) [24]. By its turn, Kauffmann et al. [25] found oxygenated sesquiterpenes (82.66%) and sesquiterpene hydrocarbons (11.05%) in the composition of the *Myrciaria plinioides* essential oil.

For accesses AC67, AC132, AC92, AC160 and AC137, the main compounds were α-muurolene (21.04%), α-longipinene (24.21%), patchoulene (32.56%), caryophyllene (48.51%) and γ-selinene (58.18%), respectively. Similar results were reported by Silva Barbosa et al. [26] by identifying the chemical composition of the essential oil of the peel of fruits of *Myrciaria floribunda*. A total of 26 compounds were identified, most of them belonging to the sesquiterpenes class—notably γ-cadinene (15.69%), γ- muurolene (6.21%), α-selinene (6.11%), α-muurolene (6.11%), caryophyllene (5.54%) and α-copaene (5.02), as they were the major compounds.

Comparing the results of the present study with those reported in fruits of the same genus (Myrciaria), there was similarity in the presence of several compounds, such as γ-muurolene, δ-elemene, α-cubebene, γ-cadinene, α-selinene, α -muurolene, aristolene, α-terpineol, eucalyptol, ocimene, and α-pinene, reported by Freitas et al. [27] and Rondán Sanabria et al. [28] in fruits of jabuticaba (*Myrciaria jabuticaba*).

In camu-camu fruits (*Myrciaria dubia*), the compound caryophyllene was identified, and presented greater abundance in the present study [18]. In the essential oil of Eugenia *involucrata* (Myrtaceae) leaves, the compounds δ-elemene, α-cubebene, γ-cadinene, α-gurjunene, and caryophyllene were reported [29].

The caryophyllene is a natural sesquiterpene found in the essential oils of various spices, fruits, and medicinal and ornamental plants [30], such as cloves (*Syzygium aromaticum*), belonging to the Myrtaceae family [31]. The caryophyllene and its derivatives have numerous properties—natural insecticide, acaricide, repellent, attractive and antifungal properties [32], anti-inflammatory, antitumor, antibacterial, antioxidant and spasmolytic [33].

In addition, the Food and Drug Administration (FDA) and the European Food Safety Authority (EFSA) have approved the use of caryophyllene in food products as a food additive, flavor enhancer, and a flavoring agent; and in the perfumery sector, as fragrance or fixative plants [30,31].

**Figure 1:**
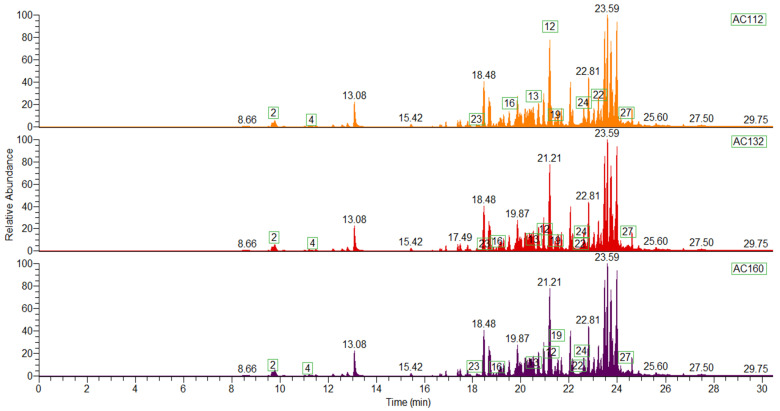
Chromatogram generated for rumberry fruits with the number of similar volatile compounds. The numbers presented in the graph corresponding to the volatile compounds arranged in Table 2 according to their numbering.

Figure 1 shows chromatograms with the largest number of compounds, differentiated by their color of orange (AC112), red (AC132) and purple (AC160), with ten of the compounds similar among them (eucalyptol, ocimene, *α*-muurolene, cyclosativene, *α*-longipinene, zonarene, eudesma-3,7(11)-diene, *α*-gurjunene, patchoulene and 10s, 11s-himachala-3 (12), 4 -diene).

Overall, the orange and red fruits did not have substantial differences when it comes to the number of compounds detected, as both had monoterpenes and sesquiterpenes in their composition. However, the purple-colored fruits showed a higher composition of sesquiterpenes than monoterpenes, notably eucalyptol.

The 1,8-cineol or eucalyptol is a monoterpene with a high therapeutic index with antihypertensive, antiasthmatic, and analgesic effects, which makes it a potent drug candidate [34]. It has biological activities such as antibacterial, antifungal, anti-inflammatory, and anti-tumor [35]. In addition, it is used in the treatment of cough, rheumatism, neurosis, muscle pain, asthma, kidney stones, and in cosmetic products.

Currently, there are no previous reports on the volatile composition of rumberry fruits. However, it is known that the Myrtaceae family commonly presents the group of sesquiterpenes as the most prevalent chemical class, and to a lesser extent, the monoterpenes [29,36]—the latter with the fungicidal and attractive function of pollinators in plants [6]. The sesquiterpenes, in turn, have shown therapeutic potential with anti-inflammatory activity in the essential oils of several plant species. Furthermore, high levels of sesquiterpenes have antioxidant properties and a wide variety of biological and pharmaceutical activities [37].

**Figure 2:**
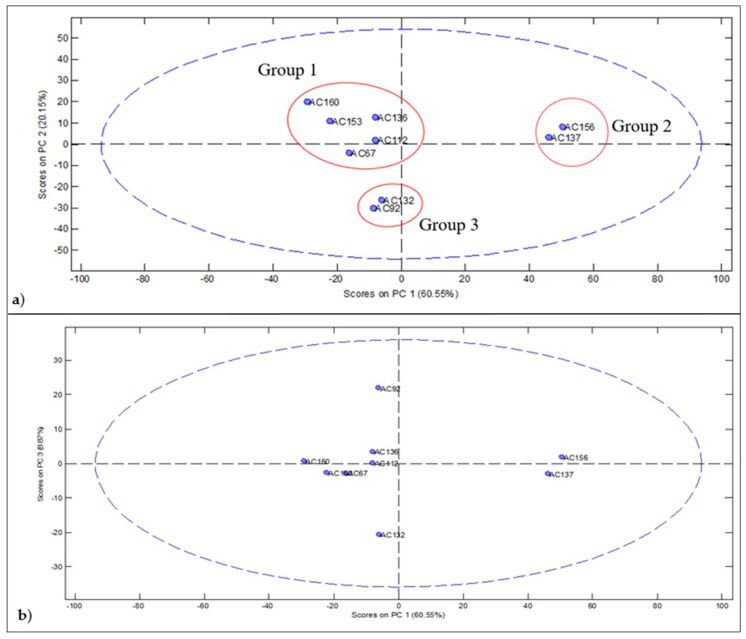
PCA scores plot obtained from the percentage of peak areas of the monoterpenes and sesquiterpenes of the volatile profile of fruits of *Myrciaria floribunda*: (**a**) groups formed with variability of 80.70% of areas; (**b**) groups formed with variability of 69.42% of the areas.

The accesses of rumberry were differentiated into three groups—AC67, AC112, AC136, AC153, and AC160 (Group 1); AC92 and AC132 (Group 2); and AC137 and AC156 (Group 3) (Figure 2). 

Group 1 (Figure 2a,b) was mainly characterized by the presence of caryophyllene (21.6% to 49.3%). However, it can be subdivided by the accesses AC112 and AC136, due to the similarity between the compounds *α*-muurolene (9.3% to 21.1) and *γ*-selinene (11.3% to 16.3), as seen in accesses AC153 and AC160, grouped by the compounds aristolene (17.0% to 19.1%) and 10s, 11s-himachala-3 (12), 4-diene (7.0% to 9.3%).

**Figure 3:**
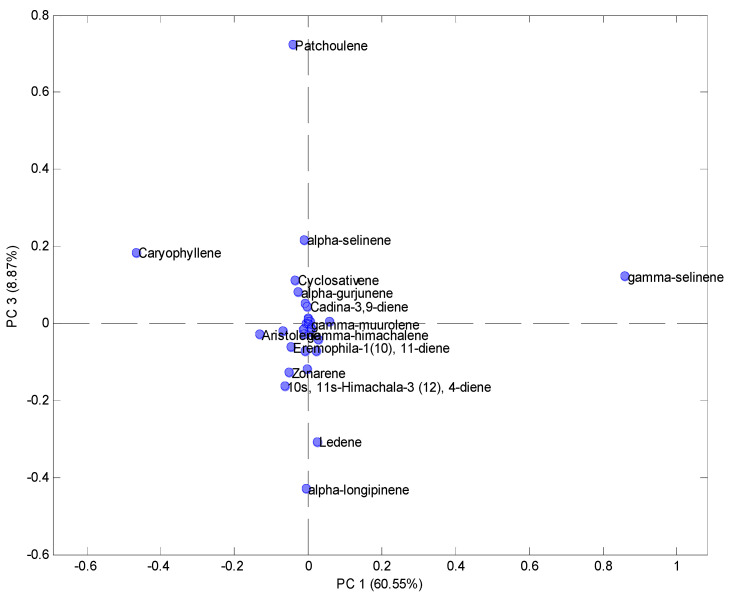
PCA loadings plot obtained from the monoterpenes and sesquiterpenes results of the volatile profile of fruits of *Myrciaria floribunda*.

Group 2 behaved differently. In Figure 2b, the interaction between main component 1 (PC1) and main component 3 (PC3) is shown. This interaction was responsible for the separation of the group by the compounds patchoulene (32.9%) and *α*-longipinene (24.9%) (Figure 3), as these compounds had the highest percentage of VOCs area identified for such accesses (Table 2). This distinction between components may occur because they are fruits of a different color.

Chalannavar et al. [38], when investigating the composition of the essential oil of *Psidium cattleianum* var. lucidum (Myrtaceae), reported patchoulene among the most predominant constituents. While Noudogbessi et al. [39] mentioned the presence of α-longipinene in the composition of the essential oil of dry leaves of *Syzygium guineense*. Li, Xuan and Shou [40] highlighted the therapeutic potential of α-longipinene against anxiety.

As for group 3, this includes the presence of the sesquiterpene γ-selinene, a compound with the highest percentage of area among the accesses (Table 2), which was similar to the subgroup composed of the accesses AC112 and AC136, as they are fruits of the same color.

Figure 3 shows the PCA loadings graph for the first three principal components. It is observed that the detailed analysis of the loadings showed in its greatest composition the class of sesquiterpenes. On the positive side of the axis of main component 1, the compounds ledene, γ-selinene, γ-muurolene, and γ-hymachalene stood out. In contrast, on the negative side of the axis were the following compounds: α-longipinene, 10s, 11s-himachala 3 (12), 4-diene, zonarene, eremophila-1 (10), 11-diene, aristolene, α-gurjunene, cyclosativene, *α*-selinene, cadina-3,9-diene, caryophyllene, and patchoulene.

It should be noted that four of the compounds present in the loadings were located at the ends of quadrant I (γ-selinene), quadrant III (α-longipinene), and quadrant VI (caryophyllene and patchoulene), due to the higher values percentage of total areas, considered as the most important compounds of the volatile profile of rumberry fruits.

**Figure 4:**
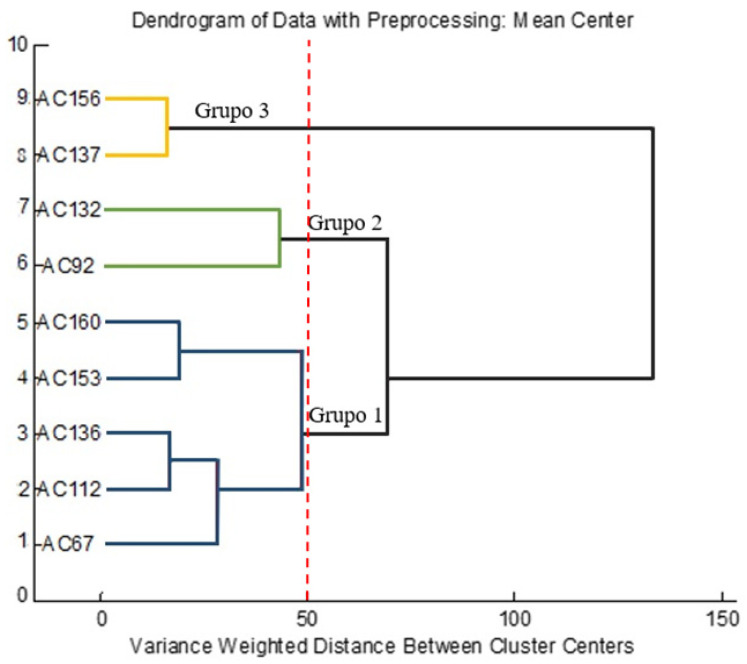
Dendrogram of the Hierarchical Cluster Analysis (HCA) of the different rumberry accesses, regarding the profile of volatile compounds.

The groups formed in the PCA (Figure 2) were confirmed with the application of the Hierarchical Cluster Analysis (HCA), considering Euclidean distances, which generated the dendrogram shown in Figure 4. The dendrogram of the Hierarchical Cluster Analysis (HCA) is presented, which grouped the rumberry accesses into three main groups with a distance = 50.

The first group was formed by the purple (AC160), the red (AC153), and the three orange accesses (AC67, AC112, and AC136) due to the high percentages of the area obtained for the caryophyllene. The second group was formed by the accesses AC92 (orange fruits) and AC132 (red fruits). However, they were isolated in the graph of scores due to their different profiles. The third group was formed by the accesses AC137 and AC156 (orange fruits) grouped mainly by the compound *γ*-selinene, which had percentage total areas of 58.9% and 66.2%, respectively.

Assessing these groups, only two (caryophyllene and γ-selinene) of the identified VOCs stood out as the main compounds responsible for the volatile profile of rumberry fruits regardless of their color, as both compounds predominated in all accesses except AC67, which did not show the presence of *γ*-selinene. Despite this, no reports were found on the volatile profile of *Myrciaria floribunda* fruits, so this is believed to be the first description of their chemical composition, so the compounds mentioned above are the first record of the species.

## 3. Materials and Methods

### 3.1. Material

Fruits of nine accesses of rumberry orchards identified by the codes AC67, AC92, AC112, AC132, AC136, AC137, AC153, AC156, and AC160 were acquired from the Cambuí Active Germplasm Bank (BAG–Cambuí), located in the municipality of Rio Largo (latitude 09°28′42″ S, longitude 35°51′12″ W, altitude of 127 m), belonging to the Center of Agricultural Sciences, of the Federal University of Alagoas (CECA-UFAL). The accesses were differentiated by the color characteristics of the fruit: orange fruits (AC67, AC92, AC112, AC136, AC137, AC156, and AC157), red fruits (AC132 and AC153), and purple fruits (AC160).

Approximately 350 g of fruits were collected per access, bagged in polyethylene bags, labeled, and packed in thermally insulated boxes with ice while being transported to the Plant Biotechnology Laboratory (BIOVEG)/CECA-UFAL. The fruits were washed in running water and sanitized with 20 mL of 1% sodium hypochlorite for 5 min. Then, a second rinse was carried out under running water for 2 min to remove chlorine residues.

Subsequently, the fruits were manually pulped, discarding only the seed and, with the help of a mixer, the pulp and the peel were homogenized. The homogenate was placed in 50 mL Falcon flasks, kept in the freezer at −60 °C until they were transported to the Mass Spectrometry Laboratory of the Department of Chemistry at the Federal University of Minas Gerais (UFMG), Belo Horizonte-MG.

### 3.2. Methods

#### 3.2.1. Physical and Physicochemical Characterization

In order to determine the physical characteristics, 128 fruits were separated by access. These fruits were individually evaluated using the weight of the fruit (FW) and the weight of the seed (SW), utilizing an analytical balance. Longitudinal diameter (LD) and transverse diameter (TD) were measured using a digital caliper. The pulp weight (PW) was obtained by the difference between the weight of the fruit and the seed. The number of seeds per fruit (NS) was determined by manual counting and the firmness obtained through a digital penetrometer with a 3.0 mm diameter tip, expressed in Newton (N).

For the physicochemical analysis, the samples (32 fruits) were crushed and homogenized with the aid of a mortar. The analyses were carried out in four replicates according to the “Analytical Norms of the Aldo Lutz Institute” [41]. The pH was obtained directly from a digital pH meter, duly calibrated with buffer solutions 4.0 and 7.0. Soluble solids (SS) were quantified in a digital refractometer, expressed in Brix, according to method 932.12 of the “Association of Official Analytical Chemists-AOAC” [42]. The titratable acidity (AT) was quantified by titrimetry; approximately 2.0 g of the pulp, diluted in 50 mL of distilled water with three drops of phenolphthalein, was titrated with a 0.1 N NaOH solution. The results were expressed in citric acid g/100 mL (%) [41]. The ratio (SS/AT) was calculated by the quotient between the values of soluble solids and titratable acidity.

#### 3.2.2. Isolation of Volatile Organic Compounds (VOCs)

The solid-phase microextraction (SPME) technique was used in the headspace (HS) mode with a polyacrylate polar fiber (PA 85 μm, supelco). According to the manufacturer’s instructions, the fiber was first conditioned in a gas chromatograph (Trace GC Ultra) with a temperature range of 220–300 °C, for 60 min.

Headspace bottles (20 mL), sealed with aluminum seal and rubber septum containing 0.5 g of sample were used. Subsequently, the vials were placed onto an aluminum block and heated to 85 °C over 5 min. After that, the fiber was exposed to the sample for 26 min in the headspace. Then, the fiber (PA) was removed from the sample, and manually inserted into the GC–MS injector. 

#### 3.2.3. Separation and Identification of Volatile Compounds (VOCs)

For the separation of VOCs, the gas chromatograph (Trace GC Ultra) was coupled to a mass spectrometer (Polaris Q) with an “ion-trap” analyzer (Thermo Scientific, San Jose, CA, USA.). A capillary column HP-5 MS (5% phenyl and 95% methylpolysiloxane), (30 m × 0.25 mm × 0.25 µm) was used for the separation. Helium was used as carrier gas with a constant flow of 1 mL/minutes (Agilent Techonolgies Inc, Waldbronn, Germany). The injector temperature was 250 °C in the splitless mode, time 5 min; the temperature of the ion source was 200 °C and the temperature of the interface temperature was 270 °C. For this, the following oven programming was used, starting at 40 °C for 5 min, with an increase of 5 °C/min until reaching 125 °C. Then, rising to 10 °C/min until reaching 245 °C, maintaining the isotherm for 3 min [43,44,45]. 

The chromatograms were extracted from the Xcalibur 1.4 software from Thermo Electron Corporation (San Jose, CA, USA.) [46]. The detection of the compounds was performed by electron impact ionization at 70 eV, in the full scan mode, identified according to their *m*/*z* ratio of 50 to 300, at a similarity level (RSI) greater than 500 [45,46]. The mass spectra were compared with the reference compounds from the NIST library (National Institute of Standards and Technology) and the data reported in the literature. For this, the percentages of the areas the VOCs obtained were analyzed by Excel version 2013 (Microsoft, Redmond, WA, USA) [47].

#### 3.2.4. Statistics

For the physical and physical-chemical characterization of the samples, an experimental design was used entirely randomized (DIC), with four replicates of thirty-two fruits for each access, totaling 128 fruits per access. The data obtained were submitted to variance analysis and the means were compared by the Scott–Knott test, at 5% probability, using the Sisvar software, version 5.7.

The Principal Component Multivariate Analysis (PCA) and Hierarchical Cluster Analysis (HCA) were performed to verify the possible correlation between rumberry accesses and the identified VOCs. In this regard, the percentages of the total areas of the chromatographic peaks of the isolated VOCs were considered as variables, which were analyzed by the MatLab program version 7.9.0.529 (Mathworks, Natick, MA, USA) [48] with the aid of the PLS Toolbox version 5.2.2 (Eigenvectors Research, Manson, WA, USA) [49]. The data obtained were organized in a matrix composed of 9 columns (accesses) and 36 rows (VOCs), which were pre-processed by self-scaling so that each variable contributed with the same weight in the analysis. In the case of HCA, Euclidean distance was used as a dissimilarity coefficient.

## 4. Conclusions

In view of the values obtained in this work, it is concluded that the fruits of rumberry (*Myrciaria floribunda*) were characterized by being acidic, explained by the low pH levels. Among the evaluated accesses, the red fruits (AC132) stood out for presenting the highest weights and dimensions. However, the orange-colored fruits had the highest pulp yields (AC156), soluble solids (AC92), the ratio of soluble solids/titratable acidity (AC137). The purple fruits (AC160) showed greater firmness and low levels of titratable acidity. 

As for the volatile profile, 36 compounds were identified, standing out from the sesquiterpenes class. The accesses AC67, AC132, AC92, AC160, and AC137 showed the highest percentages of areas corresponding to the following compounds: *α*-muurolene, *α*-longipinene, patchoulene, caryophyllene, and γ-selinene.

According to the PCA and HCA, there was chemical variability between the different accesses of *Myrciaria floribunda*, which was verified by the three groups formed. The compounds caryophyllene and γ-selinene stood out among the identified VOCs. Said compounds mainly contribute to the volatile profile of the fruits. The identification of new VOCs enables the choice and elaboration of quality products for both the industry and the consumer, thus reducing costs and bringing greater benefits, due to the demand for finding differentiated products in the market. Therefore, knowledge of the chemical composition of rumberry fruits can contribute to the expansion of chemotaxonomic studies of this genus species, as well as to the selection of new varieties.

## Figures and Tables

**Table 1:** Physical and physicochemical parameters of the nine accesses of rumberry.

Parameters												
Access	FW	SW	PW	NS	LD	TD	Format	Firmness (N)	pH	SS	TA	SS/TA
	g				mm							
AC67	0.58 a	0.17 a	0.41 a	1.18 a	8.91 b	9.95 b	0.90 b	1.46 a	3.53 a	21.30 d	5.15 b	4.15 a
AC92	0.43 a	0.14 a	0.29 a	1.19 a	7.10 a	8.94 a	0.86 a	2.59 a	3.83 a	22.78 e	5.05 b	4.51 a
AC112	0.79 b	0.25 b	0.54 b	1.71 c	10.81 d	9.81 b	1.10 d	3.26 a	3.68 a	16.88 b	4.93 b	3.60 a
AC132	1.14 c	0.49 c	0.64 b	1.66 c	11.64 e	10.43 c	1.12 d	1.44 a	3.99 a	17.65 c	4.93 b	3.58 a
AC136	0.81 b	0.21 b	0.61 b	1.48 b	10.69 d	9.65 b	1.11 d	3.57 a	3.75 a	16.53 b	4.90 b	3.55 a
AC137	0.77 b	0.16 a	0.61 b	1.50 b	10.76 d	9.54 b	1.13 d	2.61 a	3.99 a	18.30 c	2.70 a	6.87 b
AC153	0.82 b	0.24 b	0.58 b	1.42 b	9.50 b	10.47 c	0.91 b	1.98 a	3.90 a	16.10 b	4.35 b	3.79 a
AC156	0.91 b	0.22 b	0.69 b	1.91 c	10.66 d	11.50 d	0.93 c	4.08 a	3.62 a	15.43 b	3.95 b	3.98 a
AC160	0.57 a	0.10 a	0.47 a	1.01 a	9.84 c	8.82 a	1.12 d	5.67 a	3.35 a	13.25 a	2.80 a	4.75 a
Mean	0.76	0.22	0.54	1.45	10.06	9.90	1.02	2.96	3.74	17.58	4.31	4.31
CV (%)	20.07	20.83	22.43	12.11	5.09	5.95	1.51	56.17	9.83	4.02	14.66	16.28
Standard Error	0.08	0.02	0.06	0.09	1.26	0.29	0.01	0.83	0.18	0.35	0.32	0.35

Mean values of four replicates of 32 fruits per access, expressed on a wet basis. Averages followed by the same letter in the column do not differ statistically by the Scott–Knott Test, at 5% probability. FW: fruit weight; SW: seed weight; PW: pulp weight; NS: number of seeds; LD: longitudinal diameter; TD: transverse diameter; Format: the relationship between LD/TD variables; pH: hydrogen potential; SS: soluble solids (Brix); TA: titratable acidity (% citric acid); SS/TA: the ratio between the two variables. Means on the same column followed by the same letter do not differ from each other by the Scott–Knott test at the 5% probability level.

**Table 2:** Volatile profile of fruits of *Myrciaria floribunda*, isolated by the polyacrylate fiber and SPME-HS/GC–MS.

No.	VOCs	CAS	% Area								
			AC67	AC92	AC112	AC132	AC136	AC137	AC153	AC156	AC160
1	α-pinene	80-56-8	0.59	0.24	0.19	1.09	‒	0.54	‒	0.08	‒
2	Eucalyptus	470-82-6	10.6	0.29	1.26	4.36	4.04	2.45	8.74	6.56	0.7
3	3-carene	13466-78-9	5.0	1.06	0.09	1.09	2.48	1.66	1.69	‒	‒
4	Ocimene	502-99-8	1.17	1.58	0.2	4.76	0.21	0.63	0.74	0.03	0.18
5	α-terpineol	98-55-5	1	0.61	2.69	3.93	4.44	5.66	‒	‒	0.78
6	α-canfolenal	4501-58-0	0.2	0.71	0.11	0.21	0.5	‒	0.48	0.37	‒
7	Isopulegol acetate	57576-09-7	‒	1.44	0.21	1.25	0.18	0.4	2.98	1.25	‒
8	γ-terpineol	586-81-2	‒	0.29	0.15	0.47	‒	0.32	‒	0.19	‒
9	Acetate fenquila	13851-11-1	‒	0.25	0.09	‒	‒	0.56	‒	‒	‒
10	Borneol	507-70-0	‒	‒	‒	0.4	‒	1.1	‒	‒	‒
11	Isobornyl format	1200-67-5	5.83	‒	‒	‒	‒	‒	‒	0.1	‒
Monoterpenes			24.26	6.47	4.99	17.56	11.85	13.32	14.63	8.58	1.66
12	α-muurolene	10208-80-7	21.04	1.19	9.27	1.13	9.72	1.22	1.17	1.46	1.57
13	Cyclosativene	22469-52-9	3.95	9.29	6.78	4.37	0.64	0.96	0.99	0.48	2.51
14	β-guaiene		0.48	0.38	1	2.45	5.15	5.58	2.85	3.1	3.21
15	Caryophyllene		21.59	8.20	25.8	1.77	35.73	0.05	32.88	0.45	48.51
16	α-longipinene	5989-08-2	3.99	4.66	3.78	24.21	3.63	6.2	1.04	1.28	2.42
17	Longifolene	61262-67-7	6.35	3.50	4.58	4.24	0.36	‒	0.4	5.18	0.48
18	α-selinene	473-13-2	0.86	11.62	2.39	2.45	1.23	1.4	‒	‒	0.3
19	Zonarene	41929-05-9	2.11	3.38	3.43	8.92	0.37	0.47	4.5	0.51	4.81
20	γ-selinene	515-17-3	‒	4.64	11.2	0	16.05	58.18	0.4	63.1	0.63
21	Ledene	21747-46-6	0.4	0.03	4.38	13.21	0.17	7.7	0.52	‒	2.44
22	Eudesma-3,7 (11) -diene	6813-21-4	0.15	0.16	0.07	0.3	0.16	0.62	2.27	8.14	0.14
23	α-gurjunene	489-40-7	6.6	4.05	4.45	0.3	3.61	0.34	0.16	0.32	0.23
24	Patchoulene	1405-16-9	1.62	32.56	1.87	1.74	3.07	0.48	0.52	0.42	0.92
25	Eremophila-1 (10), 11-diene	10219-75-7	2.6	2.67	4.88	5.85	2.64	‒	1.69	0.05	3.94
26	γ-himachalene	53111-25-4	‒	0.05	0.34	0.77	0.25	1	4.07	0.32	1.05
27	10s, 11s-himachala-3 (12), 4-diene	60909-28-6	0.2	0.68	0.78	7.6	1.18	0.49	8.89	0.65	6.94
28	Aristolen	88-84-6	0.2	0.23	1.37	‒	1.05	0.71	18.22	0.52	16.7
29	γ-cadinene	39029-41-9	0.45	2.27	3.53	‒	0.08	0.03	‒	‒	‒
30	Cadina-3,9-diene	523-47-7	1	1.75	2.88	‒	0.83	‒	0.18	0.76	‒
31	α-cubebene	17699-14-8	0.3	0.03	0.29	‒	0.25	‒	‒	‒	‒
32	α-ylangene	14912-44-8	‒	0.19	0.38	‒	0.44	‒	‒	‒	‒
33	Guayana-1 (5), 11-diene	3691-12-1	0.11	0.52	0.29	‒	‒	0.05	‒	0.03	‒
34	δ-elemene	20307-84-0	‒	0.04	0.13	‒	‒	‒	‒	‒	‒
35	Copena	3856-25-5	1.4	0.45	0.48	0.33	‒	‒	‒	‒	‒
36	γ-muurolene	30021-74-0	‒	‒	‒	‒	‒	0.04	‒	‒	‒
Sesquiterpenes			75.42	92.54	94.35	79.64	86.61	85.52	80.75	86.77	96.8
Total identified			99.68	99.01	99.34	97.2	98.46	98.84	95.38	95.35	98.46

Accesses differentiated by the color characteristic of fruit: orange fruits (AC67, AC92, AC112, AC136, AC137, AC156, and AC157), red fruits (AC132 and AC153) and purple fruits (AC160). ‒: undetected compounds.

## Data Availability

All data are contained within the article.

## Sample Availability

Samples of the *Myrciaria floribunda* are available from the authors.
